# Supplementary material for: Unveiling the Multifaceted Role of HP6: A Critical Regulator of Humoral Immunity in Antheraea pernyi (Lepidoptera: Saturniidae)
Source: Int J Mol Sci. 2025 May 9;26(10):4514. doi: 10.3390/ijms26104514 (PMC12111086; doi:10.3390/ijms26104514)
Supplement: Supplementary file 1 [file ijms-26-04514-s001.zip › Supplementary-Figure S1-S5.pdf]

1. A volcano plot was generated to visualize differentially expressed genes in *A. pernyi* second-instar larvae subjected to microbial induction.

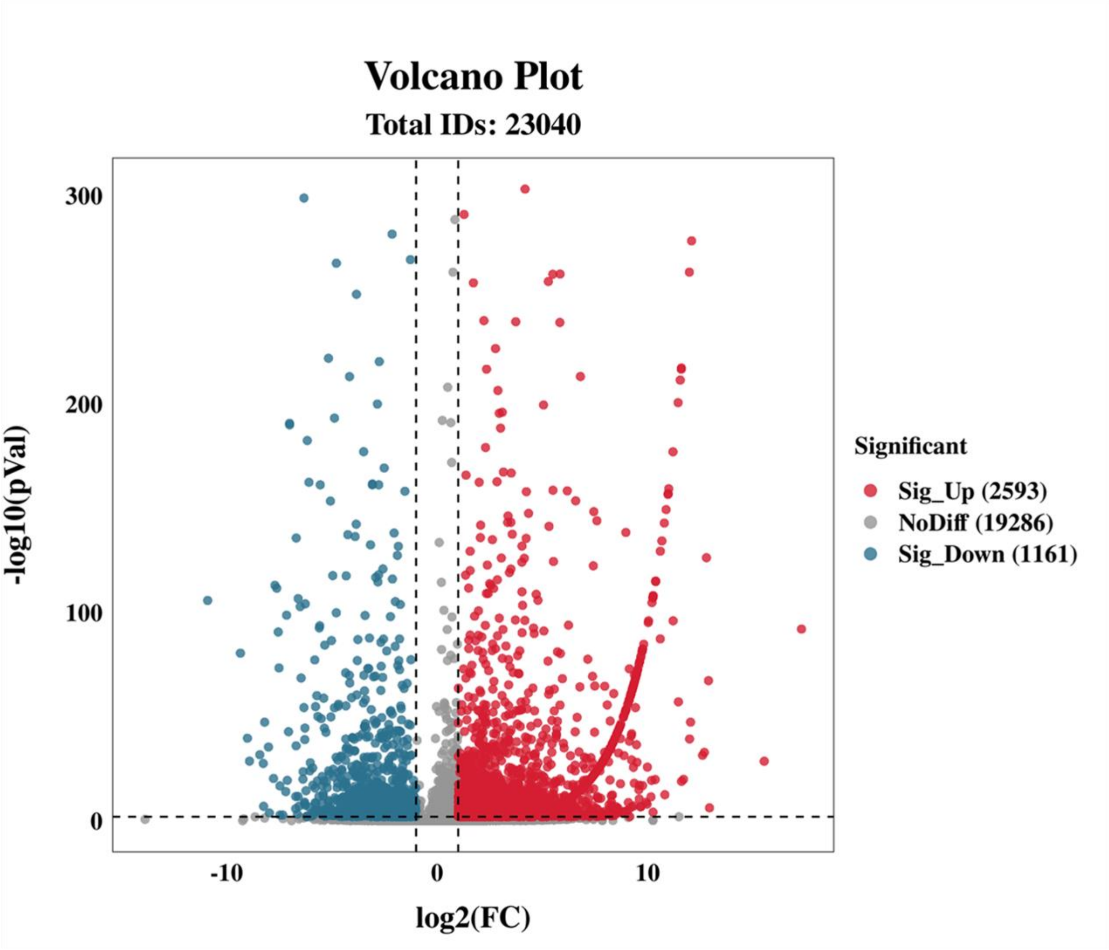

Figure S1. A volcano plot was generated to visualize differentially expressed genes in *A. pernyi* second-instar larvae subjected to microbial induction. The X-axis represents the Log_2 fold change (Log_2FC) of genes, while the Y-axis shows the negative logarithm of p-values ($-log_{10}$(p-value)). Red dots indicate significantly upregulated genes ($Log_2FC > 1$, p-value < 0.01), blue dots denote downregulated genes ($Log_2FC < -1$, p-value < 0.01), and gray dots represent non-significant genes. Dashed lines mark the thresholds for statistical significance (p-value = 0.01) and fold change ($Log_2FC = \pm 1$)

2. The relative expression level of Toll signaling pathway related factors spätzle detected by RT-qPCR.

Larvae subjected to ds-*Ap-proHP6*-treatment were injected with 5 μL suspensions of *C. albicans* and *S. aureus* (2×10^8 CFU/mL in insect saline), respectively. Control groups consisted of larvae injected with insect saline instead of microbial suspensions. Total RNA was extracted from whole larvae at 9 hr post-injection, followed by cDNA synthesis. The relative expression of spätzle (the terminal protein of the Toll signaling pathway) was quantified using RT-qPCR. Knockdown of *Ap*-proHP6 expression led to a concomitant decrease in spätzle transcript levels in both *S. aureus*- and *C. albicans*-challenged.

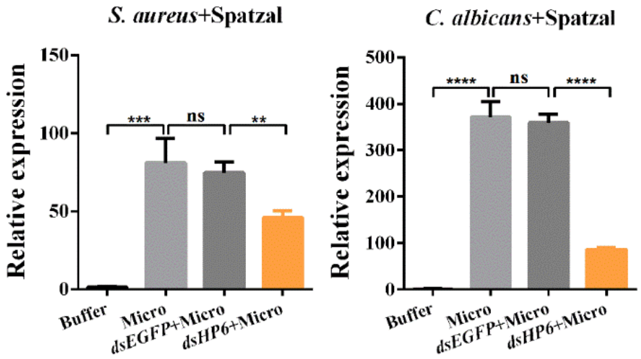

Figure S2. The relative expression level of Toll signaling pathway related factors spätzle detected by RT-qPCR. Each bar represents the mean ± SD (N=3). **: $p < 0.01$, ***p: < 0.001, ****p: < 0.0001; ns: no significant difference (Student's t-test).

3. Effect of endogenous *Ap*-proHP6 mRNA silencing on the degree of microbiota-triggered epidermal melanization of *A. pernyi*.

We examined how *Ap*-proHP6 suppression affects pathogen-induced melanization on the cuticle of fifth-instar larvae. Using RNAi, larvae injected with ds*Ap-proHP6* for 48 hr were challenged with a mixed suspension of *E. coli*, *S. aureus*, and *C. albicans*. Melanized nodules and cuticular darkening were quantified. The results showed that larvae with *Ap*-proHP6 knockdown exhibited prominent melanotic nodules and less noticeable cuticular darkening, indicating an enhanced melanization response to pathogen invasion (Figure S3).

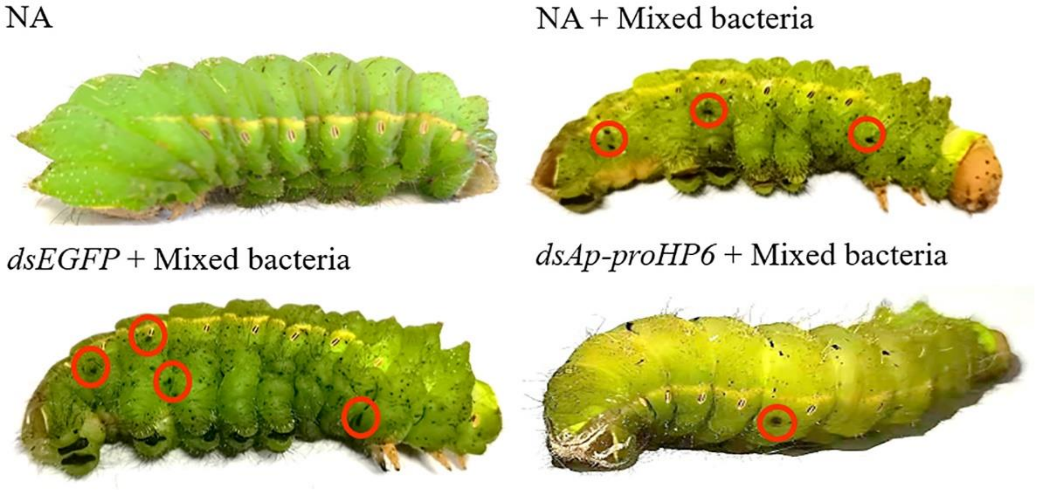

Figure S3. Effect of endogenous *Ap*-proHP6 mRNA silencing on the degree of microbiota-triggered epidermal melanization of *A. pernyi*. NA: larvae without treatment; Mixed bacteria: *E. coli*, *S. aureus*, and *C. albicans* were resuspended in insect saline and adjusted to a final concentration of 1 × 10^8 CFU/mL, and the suspensions of these three microorganisms were mixed in equal volumes; NA + Mixed bacteria: untreated larvae were injected with Mixed bacteria 48 hr later; *dsEGFP* + Mixed bacteria: larvae were injected with dsEGFP 48 hr after injection with Mixed bacteria; *dsAp-proHP6* + Mixed bacteria: larvae were injected with ds*Ap*-proHP6 48 hr after injection with Mixed bacteria

4. Determination of cleavage conditions *in vitro*

In vitro, r*Ap*-proHP6-Tb-His$_6$ was dialyzed against buffer B. Subsequently, thrombin was incubated with the protein at 23 ℃ in varying molar ratios (ranging from 1:10 to 1:100) for 6 hr. Fractions were collected, subjected to SDS-PAGE, and analyzed using ImageJ software. Similarly, *thrombin* was incubated with *Ap-proHP6-Tb-His$_6$* at different molar ratios (1:10 to 1:100) for 6 hr at 23 °C, and analyzed by non-reducing SDS-PAGE. Densitometric analysis of hydrolyzed protein bands determined that the optimal conditions for thrombin-mediated cleavage of r*Ap*-proHP6-Tb-His$_6$ were a thrombin-to-r*Ap*-proHP6-Tb-His$_6$ molar ratio of 1:70, incubated at 23 °C for 6 hr. Additionally, non-reducing SDS-PAGE analysis revealed that despite thrombin cleavage, r*Ap*-proHP6-Tb-His$_6$ remained as a single 35 kDa band.

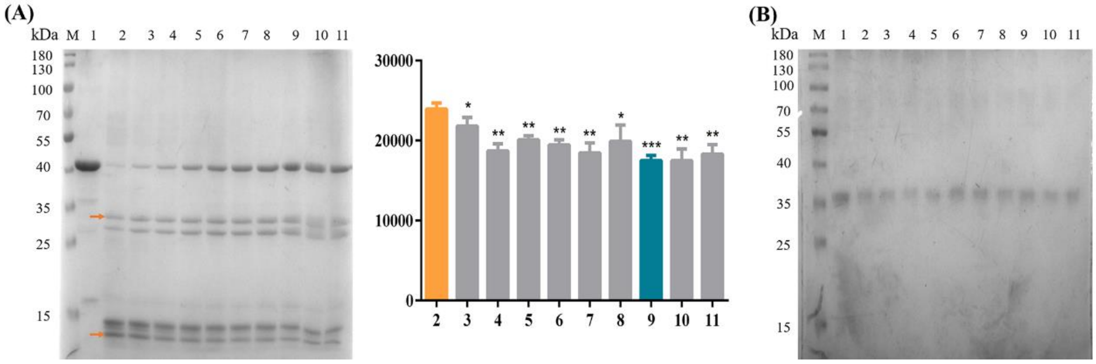

Figure S4. Conditions for thrombin cleavage of r*Ap*-proHP6-Tb-His$_6$ were determined *in vitro*. **(A)**: The in *vitro* hydrolysis efficiency of thrombin-mediated r*Ap*-proHP6-Tb-His$_6$ at varying molar ratios was evaluated using SDS-PAGE and grayscale analysis. **(B)**: The hydrolysis of r*A*p-proHP6-Tb-His$_6$ by thrombin at different molar ratios was identified by non-reducing SDS-PAGE. Lane M: Protein Marker#26616; Lane 1: *Ap*-proHP6-Tb-His$_6$ (150 µg/mL); Lane 2: Thrombin: *Ap*-proHP6-Tb-His$_6$ = 1:10; Lane 3: Thrombin: *Ap*-proHP6-Tb-His$_6$ = 1:20; Lane 4: Thrombin: *Ap*-proHP6-Tb-His$_6$ = 1:30; Lane 5: Thrombin: *Ap*-proHP6-Tb-His$_6$ = 1:40; Lane 6: Thrombin: *Ap*-proHP6-Tb-His$_6$ = 1:50; Lane 7: Thrombin: *Ap*-proHP6-Tb-His$_6$ = 1:60; Lane 8: Thrombin: *Ap*-proHP6-Tb-His$_6$ = 1:70; Lane 9: Thrombin: *Ap*-proHP6-Tb-His$_6$ = 1:80; Lane 10: Thrombin: *Ap*-proHP6-Tb-His$_6$ = 1:90; Lane 11: Thrombin: *Ap*-proHP6-Tb-His$_6$ = 1:100. *: $p < 0.05$, **: $p < 0.01$, ***p: < 0.001 (Student's t-test).

5. The experimental methodology flowchart

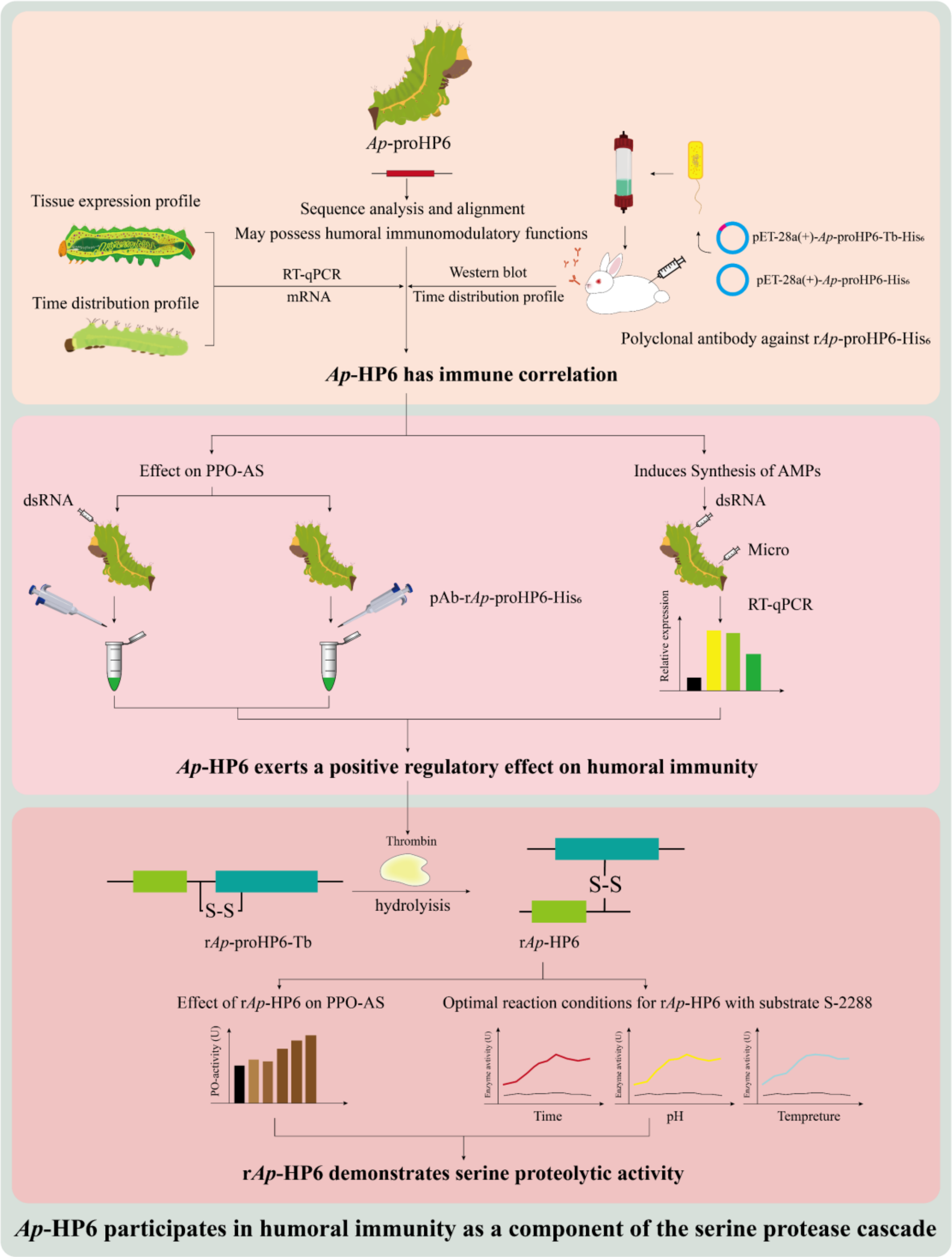

Figure S5. Flowchart of the experimental methods for investigating the active and immune function of *Ap*-HP6.
