# Supplementary material for: Unveiling the Multifaceted Role of HP6: A Critical Regulator of Humoral Immunity in Antheraea pernyi (Lepidoptera: Saturniidae)
Source: Int J Mol Sci. 2025 May 9;26(10):4514. doi: 10.3390/ijms26104514 (PMC12111086; doi:10.3390/ijms26104514)
Supplement: Supplementary file 1 [file ijms-26-04514-s001.zip › Supplementary-TABLE S1 Sequence information of primers used in the study.pdf]

TABLE S1 Sequence information of primers used in the study

Target gene		Primer name	Sequence (5′-3′)
**Primers used cDNA cloning**			
Ap-proHP6	*Nco* I	Forward	CATGCCATGGAAGATGTTGGTGAAA
	Xho I	Reverse	CCGCTCGAGTTAATTAGGCCAGACAA
Ap-proHP6-Tb	*Nco* I	Forward	CATGCCATGGGAGAAGATGTTGGTGAA<br>ATATGCAGACCAAATAATGATATA
	Not I	Reverse	TTTTCCTTTTGCGGCCGCTTAGTGATGAT<br>GGTGATGATGATTAGGCCAGACAAC
	mutant	Forward	CTGGTGCCGCGCGGCAGCGGTGGTGGT<br>GGTTCTATTATTAATGGTG
		Reverse	GCTGCCGCGCGGCACCAGGTAGAGTCC<br>AACTTTTCCTATACTATCT
**Primers used for dsRNA synthesis**			
T7-*Ap-proHP6*		Forward	TAATACGACTCACTATAGGGGTTTTATCG<br>GCAGCACACT
		Reverse	TAATACGACTCACTATAGGGCTAAAGCA<br>AGCGGTCCTC
T7-*EGFP*		Forward	TAATACGACTCACTATAGGGGCAAGGGT<br>GAGGAACTGT
		Reverse	TAATACGACTCACTATAGGGACAGCTCG<br>TCCATGCCGA
**Primers used for Real-time PCR**			
Ap-proHP6		Forward	GATTATCGTATCGTGGAA
		Reverse	CAGTAATAGTGAGAGGTG
Attacin		Forward	TGGATTGGCTTATGATAATGTC
		Reverse	GGTTGTCGTTGTGGAATAG
Defensin		Forward	TAACCATCAGCGGCAATA
		Reverse	GTTCTCCACAGTCCAAGA
Lebocin		Forward	TATTGGTGATTGCCTCAGT
		Reverse	TTTAATAACGATGGGTCTTTCC
Lysozyme		Forward	AAGCAGCCGTTATACTAAT
		Reverse	GGTGGAGGTCTTACTACA
Moricin		Forward	ATCGGATGTTAATATACAGTAAGT
		Reverse	ACACAATAAAGCACAGCAA
Spätzle		Forward	AAATTGGGCTTCTGCGAAT
		Reverse	TCTGGTGTGTCAGGTAAATCCA
18s rRNA		Forward	CGATCCGCCGACGTTACTAC
		Reverse	GTCCGGGCCTGGTGAGATT

T7 promoter sequences were underline
